# Development and validation of a nomogram for predicting recurrence-free survival in endometrial cancer: a multicenter study

**DOI:** 10.1038/s41598-023-47419-8

**Published:** 2023-11-20

**Authors:** Yinuo Li, Xin Hou, Wei Chen, Shixuan Wang, Xiangyi Ma

**Affiliations:** 1 Department of Obstetrics and Gynecology, Tongji Hospital, Tongji Medical College, Huazhong University of Science and Technology, Wuhan, Hubei China; 2 Department of Computer Center, Tongji Hospital, Tongji Medical College, Huazhong University of Science and Technology, Wuhan, Hubei China

**Keywords:** Cancer, Risk factors

## Abstract

Recurrence is the main cause of death in patients with endometrial cancer (EC). This study aimed to construct and validate a nomogram to predict the recurrence-free survival of patients with EC. This was a multicenter retrospective study. A total of 812 patients from Wuhan Tongji Hospital were divided into training and validation cohorts, and 347 and 580 patients from People’s Hospital of Peking University and Qilu Hospital of Shandong, respectively, were used for validation. Univariate and multivariate Cox regression analyses were used to construct a nomogram for predicting recurrence-free survival of EC. Calibration curves, receiver operating characteristic (ROC) curves, and consistency indexes (C-indexes) were used to estimate the performance of the model. Decision curve analysis (DCA) curves were used to assess the clinical utility of the model. Age (*P* = 0.013), cancer antigen 125 level (*P* = 0.014), lymphovascular space invasion (*P* = 0.004), International Federation of Gynecology and Obstetrics stage (*P* = 0.034), and P53 (*P* < 0.001) were independently associated with recurrence, and we constructed a nomogram based on these variables. The C-indexes of the validation cohorts were 0.880, 0.835, and 0.875, respectively. The calibration, ROC, and DCA curves revealed that this model had excellent performance and clinical utility. Combining clinical data, clinicopathological factors, serological indicators, and immunohistochemical marks, a multicenter externally verified nomogram with robust performance was constructed to predict the recurrence of patients with EC.

## Introduction

Endometrial cancer (EC) is one of the three most common gynecological malignancies and the sixth most frequent cancer in women. In 2020, 417,367 patients were newly diagnosed with EC globally, and 97,370 died^1^. Over the past decades, diagnostic methods and treatment strategies for EC have continuously improved. The majority of patients with EC receive timely surgical treatment due to diagnosis at an early stage, resulting in a good prognosis^2,3^. However, some patients still experience recurrence, which is the main cause of death in such patients^4^. The prognosis of these patients has not improved due to lack of good methods to predict recurrence^5^. For patients at high-risk for recurrence, appropriate local or systemic adjuvant therapy after surgery can reduce the incidence of recurrence and adverse complications and improve their quality of life^6,7^. Accurate assessment of the risk of recurrence of EC is crucial to both the selection of the preoperative plan and postoperative care. It avoids unnecessary suffering due to overtreatment in low-risk women, and ensures that appropriate adjuvant therapy can be administered to improve survival.

For a long time, the risk assessment of EC recurrence was based on clinicopathological parameters, including histological type, histologic grade, International Federation of Gynecology and Obstetrics (FIGO) stage, and lymphovascular space invasion (LVSI)^8,9^. There is emerging evidence that serum cancer antigen 125 (CA125)^10,11^, immunohistochemical markers, such as estrogen receptor (ER), progesterone receptor (PR), P53, and Ki67^12,13^, and some immune-inflammatory markers such as the peripheral blood neutrophil–lymphocyte ratio (NLR), platelet-lymphocyte ratio (PLR), and hemoglobin are associated with EC prognosis^14,15^. Some predictive models for the prognosis of EC have been developed^12,16^, however, few studies considered comprehensive factors to evaluate their predictive effectiveness, and some models lack external validation.

A nomogram is a computational tool that quantifies the risks of various factors^17^, and makes personalized assessments of patients, which is conducive to clinical decision-making. In this study, we collected comprehensive data from three hospitals to construct a nomogram to predict the recurrence-free survival (RFS) in EC and to perform a multicenter external validation that provides reliable evidence for physicians to make individualized clinical decisions.

## Material and methods

### Study population

The study population consisted of patients from Wuhan Tongji Hospital used for model building and preliminary validation, and patients from People’s Hospital of Peking University and Qilu Hospital of Shandong University used for external validation. We enrolled patients who underwent hysterectomy (with or without adnexectomy and lymphadenectomy) for primary EC at the Tongji Hospital, Tongji Medical College, Huazhong University of Science and Technology (Wuhan, China) between 2012 and 2020. Additionally, we enrolled patients who underwent surgery for EC between 2006 and 2018 at the People’s Hospital of Peking University and Qilu Hospital of Shandong University. Three hospitals enrolled patients according to the same criteria.

The inclusion criteria were as follows: patients who (1) underwent hysterectomy for EC; (2) had complete medical records; and (3) had follow-up information.

The exclusion criteria were as follows: (1) mixed carcinoma; (2) unclassified carcinoma; (3) EC combined with other malignant tumors; (4) death from other diseases; (5) EC diagnosed by biopsy alone, without surgical resection; and (6) lost to follow-up.

### Treatment and follow-up

All patients who were included underwent hysterectomy (with or without adnexectomy and lymphadenectomy). Adjuvant therapy (including chemotherapy, brachytherapy, external beam radiotherapy) was determined by multidisciplinary treatment according to international NCCN guidelines. Patients were followed-up every 3 months for the first 2 years after surgery, every 6 months for 3–5 years, and once a year thereafter. Follow-up content included regular gynecological examination and necessary auxiliary inspection, including serum tumor markers (such as CA125 and human epididymis protein 4) and imaging examinations (such as gynecological B-mode ultrasound, abdominal and pelvic computed tomography). The endpoint was EC recurrence. Recurrence in patients from three hospitals was recorded, including the time, location, and survival. RFS refers to the time from primary surgery to disease recurrence.

### Data collection

Eligible patients were selected based on the inclusion and exclusion criteria, and variables were collected from these patients. Candidate variables included clinically relevant variables, such as age at surgery, weight, height, and body mass index (BMI). Traditional pathological factors include histological type, tumor grade, LVSI status, presence of lymph node metastasis (LNM), cervical stromal involvement, and FIGO stage. Immunohistochemical indicators, such as ER, PR, P53, and Ki67. Hematological indicators included CA125, neutrophils, platelets, lymphocytes, and hemoglobin. If the variable was missing, it was regarded as an invalid variable and not included in the analysis and final model construction. According to the above principles, the variables included in the analysis were age, CA125, histological type, tumor histological grade, LVSI, LNM, cervical stromal involvement, FIGO stage, ER, P53, Ki67, and immune-inflammatory markers, including PLR and hemoglobin. BMI, PR, and NLR were excluded because of missing data.

### Statistical analysis

Statistical analysis was performed using R 4.1.2 software (http://www.Rproject.org). To develop an exportable and well-calibrated nomogram for RFS, the data from Wuhan Tongji Hospital were randomly divided into training and validation cohorts at a ratio of 7:3.

In the training cohort, univariate Cox proportional hazard models were used to evaluate the effects of relevant factors, including age, PLR, hemoglobin, CA125, histologic type, histologic grade, LVSI, LNM, cervical stromal invasion, FIGO, ER, P53, and Ki67. With the exception of PLR, hemoglobin, and Ki67, which were continuous variables, all other indicators were binary variables. For continuous variables, the mean and SD were used for normal distribution, and the median (range) is used if the distribution is skewed. Statistical significance was set at *P* < 0.05, and significant variables were included in the multivariable Cox regression analyses. Hazard ratios (HRs) and 95% confidence intervals (CIs) were calculated. Among these factors, only those with statistical significance were incorporated into the nomogram. Subsequently, external verification of the nomogram was performed using the validation cohort of Wuhan Tongji Hospital and the data from two other hospitals. Receiver operating characteristic (ROC) curves were used to estimate the performance based on the areas under the curves. A calibration curve was generated to determine whether the predicted probabilities tallied with the observed outcome frequencies or not. The consistency index (C-index), which varies between 0 and 1, was also used to evaluate the discernible capacity. If the C-index is > 0.7, the nomogram has a good prognostic significance. Moreover, the larger the C-index, the better the prognostic prediction. Decision curve analysis (DCA)was performed to evaluate the clinical utility.

### Ethical approval and consent to participate

This retrospective study was approved by the Institutional Review Committee of Tongji Hospital, Tongji Medical College, Huazhong University of Science and Technology (TJ-IRB20220556). Written informed consent from the patients to participate in this study was obtained before the investigation. The study was performed in compliance with the Declaration of Helsinki.

## Results

### Patients’ basic information

**Figure 1:**
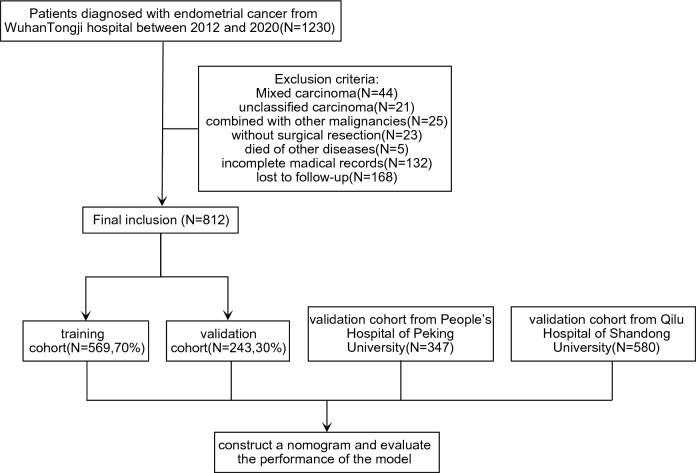
Flow chart of the study.

In total, 1230 patients with primary EC who underwent hysterectomy were retrospectively enrolled from Wuhan Tongji Hospital between 2012 and 2020. Following the exclusion of some patients, 812 patients were included in the analysis, and were subsequently divided into training and validation cohorts using a 7:3 ratio for modeling and preliminary validation. Two external cohorts of 347 patients from Peking University People's Hospital and 580 from Shandong University Qilu Hospital were used to verify the performance of the model. The flowchart is shown in Fig. 1. Basic information on several cohorts of patients, according to the included indicators are presented in Table 1. The major histological subtype was type I, regardless of the hospital. Type I EC, known as endometrioid EC, is estrogen-dependent and mainly composed of endometrial gland-like tissue. Type II EC, known as non-endometrioid EC, is non-estrogenic-dependent and includes serous and clear cell carcinomas. Additionally, most patients were diagnosed with FIGO I or II. There were very few patients in each hospital who had not undergone lymph node dissection; therefore, we defined these patients as having an unknown LNM status, which is also in line with the actual clinical situation. For P53 immunohistochemistry, each hospital was evaluated by two independent pathologists.

**Table 1:** Baseline characteristics of the training and validation cohorts.

Variable	Training cohort, N = 569	%	Validation cohort from Tongji Hospital, N = 243	%	Validation cohort from People’s Hospital of Peking University, N = 347	%	Validation cohort from Qilu Hospital of Shandong University, N = 580	%
Recurrence								
No	522	91.7	223	91.8	315	90.8	559	96.4
Yes	47	8.3	20	8.2	32	9.2	21	3.6
Age								
< 60	437	76.8	194	79.8	236	68.0	438	75.5
≥ 60	132	23.2	49	20.2	111	32.0	142	24.5
PLR								
Median (range)	134.64 (33.33–492.78)		137.44 (44.30–404.12)		154.64 (6.08–1451.72)		152.10 (2.73–836.36)	
Homoglobin								
Median (range)	124.00 (49.00–159.00)		123.00 (47.00–162.00)		121.90 (68.00–160.00)		130.00 (4.00–284.00)	
CA125								
< 35	444	78.0	179	73.7	258	74.4	456	78.6
≥ 35	125	22.0	64	26.3	89	25.6	124	21.4
Histologic type								
Type I	544	95.6	230	94.7	316	91.1	553	95.3
Type II	25	4.4	13	5.3	31	8.9	27	4.7
Histologic grade								
1	278	48.9	108	44.4	110	31.7	297	51.2
2	189	33.2	85	35.0	173	49.9	193	33.3
3	102	17.9	50	20.6	64	18.4	90	15.5
Cervical stromal invasion								
No	518	91.0	218	89.7	305	87.9	519	89.5
Yes	51	9.0	25	10.3	42	12.1	61	10.5
LVSI								
No	541	95.0	226	93.0	299	86.2	532	91.7
Yes	28	5.0	17	7.0	48	13.8	48	8.3
LNM								
No	456	80.1	191	78.6	295	85.0	523	90.2
Yes	43	7.6	17	7.0	28	8.1	25	4.3
Unknown	70	12.3	35	14.4	24	6.9	32	5.5
FIGO								
I-II	490	86.1	210	86.4	295	85.0	525	90.5
III-IV	79	13.9	33	13.6	52	15.0	55	9.5
ER								
+	513	90.2	214	88.1	321	92.5	537	92.6
−	56	9.8	29	11.9	26	7.5	43	7.4
P53								
Wild	455	80.0	206	84.8	155	44.7	428	73.8
Mutation	114	20.0	37	15.2	192	55.3	152	26.2
Ki67								
Median (range)	0.40 (0.01–0.90)		0.40 (0.03–0.90)		0.38 (0.02–0.95)		0.34 (0.01–0.95)	

*PLR* platelets/lymphocytes, *LVSI* lymphovascular space invasion, *LNM* lymph node metastasis, *ER* estrogen receptor.

### Univariate and multivariate predictors for prognostic factors

Univariate and multivariate Cox proportional hazards models were used to select independent risk factors (Tables 2 and 3). According to the univariate Cox proportional hazard model, age (*P* = 0.006), CA125 (*P* < 0.001), histological grade (*P* < 0.001), LVSI (*P* < 0.001), FIGO (*P* < 0.001), ER (*P* = 0.026), and P53 (*P* < 0.001) were all significantly associated with recurrence. However, the multivariate Cox proportional hazard model revealed that histologic grade and ER were not significantly associated with recurrence; therefore, these two indicators were excluded from subsequent modeling. The remaining five variables, including age (HR: 2.12; 95% CI 1.17 to 3.83; *P* = 0.013), CA125 (HR: 2.19; 95% CI 1.71 to 4.11; *P* = 0.014), LVSI (HR: 3.55; 95% CI 1.51 to 8.36; *P* = 0.004), FIGO (HR: 2.09; 95% CI 1.06 to 4.11; *P* = 0.034), and P53 (HR: 3.09; 95% CI 1.67 to 5.73; *P* < 0.001) were independently associated with recurrence and finally included in the model. Among the remaining factors, LVSI was the most predictive factor for prognosis; patients with LVSI had a 3.55-fold increased risk of recurrence. Besides, P53 mutations were also important for predicting the probability of recurrence in patients with endometrial cancer, and the representative images of immunohistochemistry were shown in Fig. S1.

**Table 2:** Univariate Cox proportional hazard models of predictive factors.

Variable	Hazard ratio	95%CI	P value
Age			
< 60 vs ≥ 60	2.282	1.274–4.089	0.006
PLR	1.001	0.996–1.005	0.811
Hemoglobin	0.997	0.982–1.012	0.655
CA125			
< 35 vs ≥ 35	2.982	1.667–5.333	< 0.001
Histologic type			
Type I vs type II	2.097	0.751–5.849	0.157
Histologic grade			
1	1		
2	2.293	1.424–3.692	< 0.001
3	1.371	0.813–2.312	0.236
LVSI			
No vs yes	4.897	2.176–11.018	< 0.001
LNM			
No vs yes	0.59	0.259–1.344	0.209
Cervical stromal invasion			
No vs yes	2.019	0.943–4.322	0.071
FIGO			
I II vs III IV	3.715	2.025–6.817	< 0.001
ER			
Positive vs negative	2.287	1.105–4.730	0.026
P53			
Wild vs mutation	4.074	2.273–7.305	< 0.001
Ki67	2.662	0.736–9.624	0.135

*PLR* platelets/lymphocytes, *LVSI* lymphovascular space invasion, *LNM* lymph node metastasis, ER, estrogen receptor.

**Table 3:** Multivariate Cox proportional hazard models of predictive factors.

Variable	Hazard ratio	95%CI	P value
Age			
< 60 vs ≥ 60	2.116	1.169–3.831	0.013
CA125			
< 35 vs ≥ 35	2.192	1.71–4.105	0.014
Histologic grade			
1	1		
2	1.17	0.067–2.041	0.58
3	1.212	0.715–2.054	0.476
LVSI			
No vs yes	3.547	1.506–8.355	0.004
FIGO			
I II vs III IV	2.085	1.058–4.106	0.034
ER			
Positive vs negative	0.985	0.443–2.189	0.97
P53			
Wild vs mutation	3.088	1.666–5.725	< 0.001

*LVSI* lymphovascular space invasion, *ER* estrogen receptor.

### Nomogram development in the training cohort

**Figure 2:**
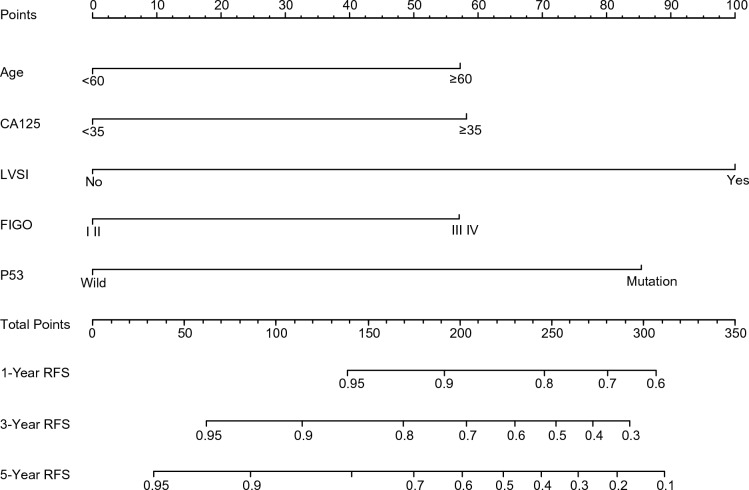
Nomogram for predicting the 1-, 3-, and 5-year probabilities of RFS in EC. Draw a straight line for each factor measured in patients to the axis labeled “Points.” Add up these scores to get a total score and then draw a vertical line to the bottom scale to get the predicted probability. EC: Endometrial cancer; RFS: Recurrence-free survival.

The patients from Wuhan Tongji Hospital were randomly divided into a training set (*n* = 569) and validation set (*n* = 243) using the R 4.1.2 software. To provide quantitative measurement for better prediction of recurrence, a nomogram analysis of RFS was constructed by the Cox proportional hazard models using five statistically significant indicators (Fig. 2). As shown in the nomogram, the predictive value of each predictor corresponded to the line segment length. For individualized prediction, add up the scores for each characteristic of the patient by drawing a vertical line upward to the “Points” line and get the total points. Drawing a straight line down to the bottom scale allowed the 1-, 3-, and 5-year probabilities of RFS to be predicted. In addition, we have generated a calculator online that predicted RFS of EC for open accessing and provide the link at the end of the article.

### Evaluating the performance of the nomogram

**Figure 3:**
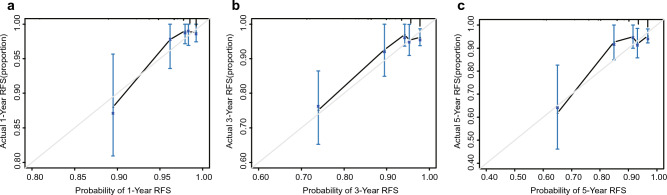
The calibration curve of the nomogram model. The grey line is the ideal line on which the data would lie. The black line represents the actual predicted performance of the nomogram. The vertical lines indicate 95% confidence intervals. (**a**) The calibration curve for the nomogram of predicting 1-year RFS in EC; (**b**) The calibration curve for the nomogram of predicting 3-year RFS in EC; (**c**) The calibration curve for the nomogram of predicting 5-year RFS in EC. EC: Endometrial cancer; RFS: Recurrence-free survival.

**Figure 4:**
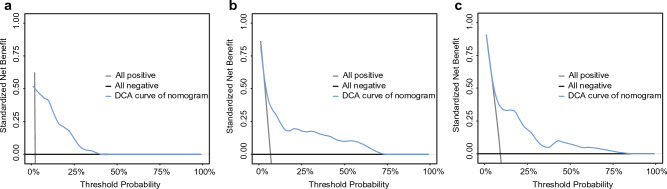
The DCA curve of the nomogram model. The all-positive line represents that all patients survived. The all-negative line represents that no patients survived. The DCA curve of nomogram shows the clinical net benefits. (**a**) The DCA curve for the nomogram of predicting 1-year RFS in EC; (**b**) The DCA curve for the nomogram of predicting 3-year RFS in EC; (**c**) The DCA curve for the nomogram of predicting 5-year RFS in EC. DCA: Decision curve analysis; EC: Endometrial cancer; RFS: Recurrence-free survival.

**Figure 5:**
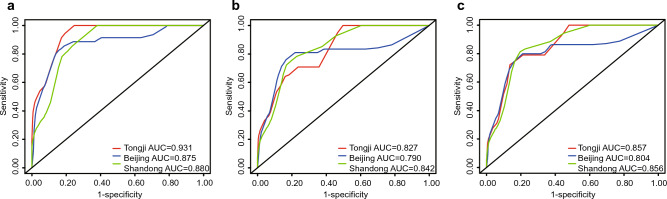
The ROC curve analysis of the nomogram. The red line represents the validation cohort from Tongji Hospital. The blue line represents the validation cohort from People’s Hospital of Peking University. The green line represents the validation cohort from Qilu Hospital of Shandong University. (**a**) The ROC curve of the nomogram to predict 1-year RFS in EC; (**b**) The ROC curve of the nomogram to predict 3-year RFS in EC; (**c**) The ROC curve of the nomogram to predict the 5-year RFS in EC. EC: Endometrial cancer; RFS: Recurrence-free survival; ROC: Receiver operating characteristic.

In the training cohort, calibration curves were generated to evaluate the accuracy of the nomograms, which showed ideal prediction ability at 1, 3, and 5 years for RFS versus actual probability (Fig. 3). We also performed the calibration curves from three validation cohorts that described the association between probability and actual RFS (Figs. S2–S4). The calibration curves from all cohorts indicated that the model had high accuracy. DCA was performed to evaluate clinical utility. As shown in Fig. 4, DCA satisfactorily predicted 1-, 3-, and 5-year RFS. In addition, in validation cohorts, the ROC curve and C-index more intuitively revealed the prediction performance of the nomogram (Fig. 5). The 1-, 3-, and 5-year area under the curve values of RFS from the Wuhan Tongji Hospital were 0.931, 0.827, and 0.857, respectively. The other two validation cohorts also revealed good efficacy in predicting prognosis. We used total points from the nomogram as an independent variable in the ROC analysis. We calculated the Youden index to obtain the optimal cutoff values (Table S1). The C-indexes of the nomogram were 0.880, 0.835, and 0.875 for the three cohorts, respectively (Table 4).

**Table 4:** The predictive performance of nomogram (AUC and C-index).

Cohort	1-year AUC	3-year AUC	5-year AUC	C-index (95%CI)
Validation cohort from Tongji Hospital	0.931	0.827	0.857	0.880 (0.813–0.948)
Validation cohort from People’s Hospital of Peking University	0.875	0.790	0.804	0.835 (0.752–0.917)
Validation cohort from Qilu Hospital of Shandong University	0.880	0.842	0.856	0.875 (0.811–0.940)

## Discussion

In recent years, the incidence of EC has continuously increased, but the 5-year survival rate of patients with EC has not improved significantly^18^. At present, either postoperative radiotherapy or chemotherapy is selected according to FIGO stage and traditional pathological risk factors, however, the efficacy is not satisfactory^19–21^. The Cancer Genome Atlas molecular typing of EC is an accurate risk stratification method to evaluate prognosis, but is difficult to popularize in clinical practice at present^22,23^. In the absence of comprehensive prognostic assessment tools, the optimal therapy for patients with high-risk EC is difficult to identify^5^. In our study, we developed a nomogram that is easy to operate based on the results of multivariate Cox regression analyses. Variables including age ≥ 60 years, CA125 ≥ 35 IU/mL, LVSI, P53 mutation, and advanced FIGO stage were independent risk factors of the recurrence of EC. The C-index of the nomogram constructed using these factors reached 0.880 in the Tongji validation, indicating excellent effectiveness. The C-indexes of this model in the cohorts from the other two centers were 0.835 and 0.875, respectively, which proves that its robustness.

Previous studies constructed models to predict the prognosis of EC. Ouldamer et al.^16^ developed a nomogram to predict the likelihood of a poor 3-year prognosis for recurrence in patients with EC. Although the AUC was 0.82, they only considered clinical and histological variables. Some immunohistochemistry-based markers were proven to be useful tools for EC risk stratification. Nomograms based on immunohistochemical markers and clinical parameters were developed and revealed good effectiveness^24^. However, these models can be further optimized. Chronic inflammation is one of the underlying mechanisms of carcinogenesis, including EC^25,26^. Some studies have shown that inflammation is associated with the prognosis of cancer. Some immune-inflammatory markers, such as NLR, PLR, and hemoglobin, have been widely used to predict the prognosis of various cancers, such as lung cancer^27,28^, breast cancer and esophageal squamous cell carcinoma^29,30^. Cong et al.^14^ discovered that a model combining NLR, PLR, monocyte-lymphocyte ratio, and clinicopathological factors can improve the accuracy of predicting the overall survival. However, few studies have taken into account basal clinical features, clinicopathological parameters, immunohistochemical markers, and immunoinflammatory markers to assess their association with prognosis of EC. We considered these factors comprehensively and finally included five statistically significant risk factors to build the model. In addition, although the two external validation cohorts were from other hospitals, the validation results were good, which confirms the wide application of this model.

In comparison with previous studies, multivariate analysis showed that serum CA125 levels were significant, and serum CA125 was shown to be associated with the recurrence of EC; therefore, the preoperative CA125 level should not be underestimated when it is elevated above normal. In our study, we classified CA125 according to a binary classification, which is based on the clinically recognized and widely used CA125 cutoff value of 35 IU/mL, for EC^31,32^. In addition, the contribution of P53 in the model is also very prominent. P53 immunohistochemistry predicts TP53 mutation in endometrial carcinoma^33^ and TP53 mutation is a marker of high copy number abnormalities (CN-high), which represents a poor prognosis in The Cancer Genome Atlas molecular subtype^34^. Our results also revealed that p53 mutations are closely related to the recurrence of endometrial carcinoma. Compared to sequencing, p53 immunohistochemistry is inexpensive and easy to perform; therefore, it is more practical in clinical settings. Moreover, in our model, LVSI had the highest HR, which indicates that it is closely related to the recurrence of EC. Numerous studies reported that LVSI is associated with lymph node and distant metastases of EC^35,36^. Song et al. demonstrated that LVSI was the strongest independent factor for recurrence in a multivariate analysis^37^. Furthermore, many studies have shown that age is significantly associated with the occurrence of EC; post operative radiation therapy for EC used ≥ 60 years as a cutoff to incorporate age into the risk stratification for EC^38^. In this study, we marked 60 years as the dividing line for the two classification variables. We classified FIGO stages I and II into early stage and III and IV into advanced stage, which can improve the efficiency of the model and simplify it. Some other pathological factors, such as histological grade, lymph node metastasis, and cervical stromal invasion were not included in the model, but it is undeniable that they still have a certain impact on prognosis.

At present, surgery, supplemented by radiotherapy and chemotherapy, is the mainstay of treatment for EC. Treatment is a decision made by clinicians for patients according to NCCN guidelines. Our research focuses on indicators that can be obtained during surgery. Based on these indicators, we can predict recurrence in patients, help clinicians decide subsequent adjuvant treatment regimens, and optimize patient follow-up management.

To the best of our knowledge, this model is currently the most effective for predicting RFS in EC, and this is a multicenter study with a large number of participants. Our model achieved excellent performance in both internal and external validation cohorts, which proves that it has good applicability in the general population. It also includes clinical case data, traditional clinicopathological factors, immunohistochemical marks, and serological indicators, making it comprehensive for evaluation indicators.

However, our study had some limitations. First, as this was a retrospective study, there may be selection and recall bias, and prospective data must be collected to further verify the performance of the model. Second, the p53 status in our model cannot fully represent high copy number abnormalities, which needs to be further verified by experimental research. Furthermore, we included only four immunohistochemical markers, thus, the inclusion of some other proven indicators in the future, such as phosphatase and tensin and L1 cell adhesion molecule^39^, may further optimize the model. We will continue to validate the model with foreign cohorts to eliminate the influence of ethnic differences. In addition, we hope to find the key molecules driving recurrence by gene sequencing and to incorporate them into the model to achieve early prediction.

## Conclusion

In summary, we developed a nomogram to predict the RFS of patients with surgically treated EC, and the nomogram performed well in two other external validation cohorts. It can be used as an additional tool to better identify patients who require adjuvant treatment. Our model can also facilitate the design of clinical trials for follow-up treatment of EC by selecting high-risk groups for recurrence among women with EC.

### Supplementary Information

Supplementary Figure S1.

Supplementary Figure S2.

Supplementary Figure S3.

Supplementary Figure S4.

Supplementary Table S1.

Supplementary Legends.

## Data Availability

The link of calculator that predicted RFS of EC for open accessing is https://figp2023.shinyapps.io/EC-PredictRFS/. The other datasets generated during the current study are available from the corresponding author on reasonable request.
