# Supplementary figures and images for: Development and validation of a nomogram for predicting recurrence-free survival in endometrial cancer: a multicenter study
Source: Sci Rep. 2023 Nov 20;13:20270. doi: 10.1038/s41598-023-47419-8 (PMC10662280; doi:10.1038/s41598-023-47419-8)

FigureS1

a

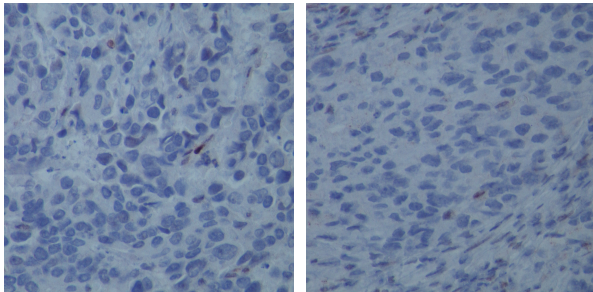

fully negative

b

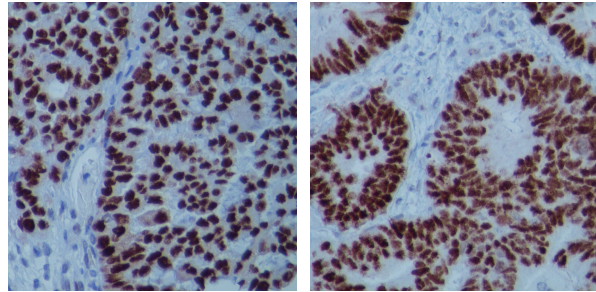

strongly positive

Supplement: Supplementary file 1 — Supplementary Figure S1. [file 41598_2023_47419_MOESM1_ESM.pdf]

FigureS2

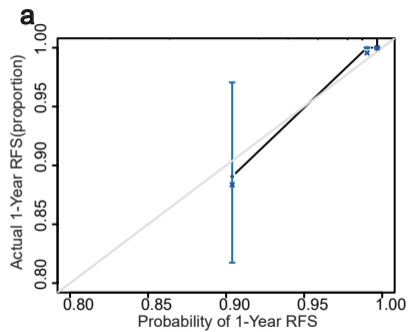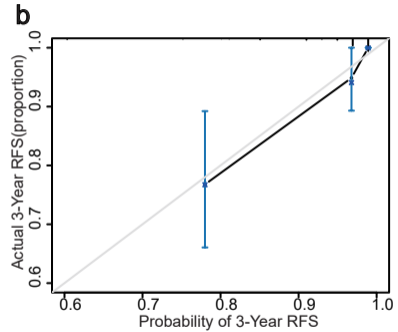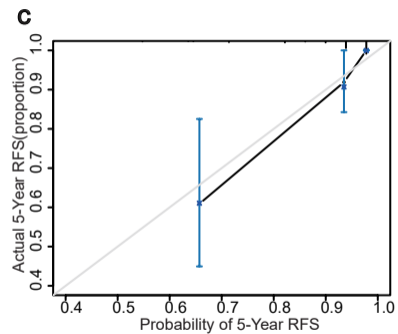

Supplement: Supplementary file 2 — Supplementary Figure S2. [file 41598_2023_47419_MOESM2_ESM.pdf]

FigureS3

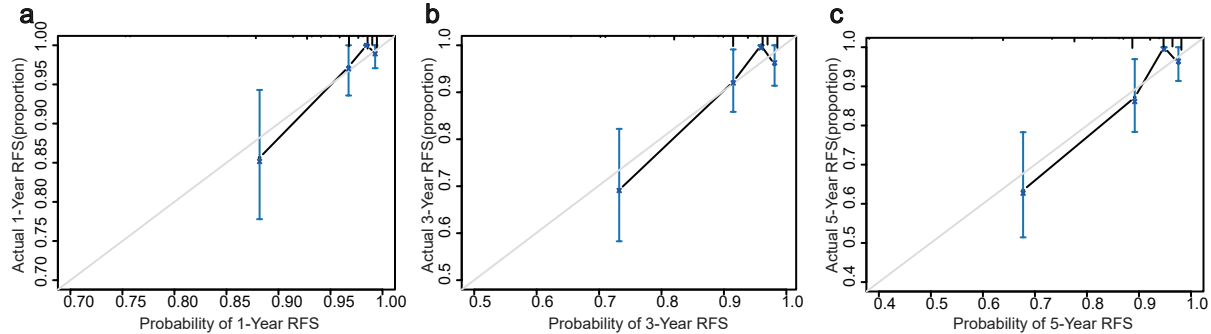

Supplement: Supplementary file 3 — Supplementary Figure S3. [file 41598_2023_47419_MOESM3_ESM.pdf]

FigureS4

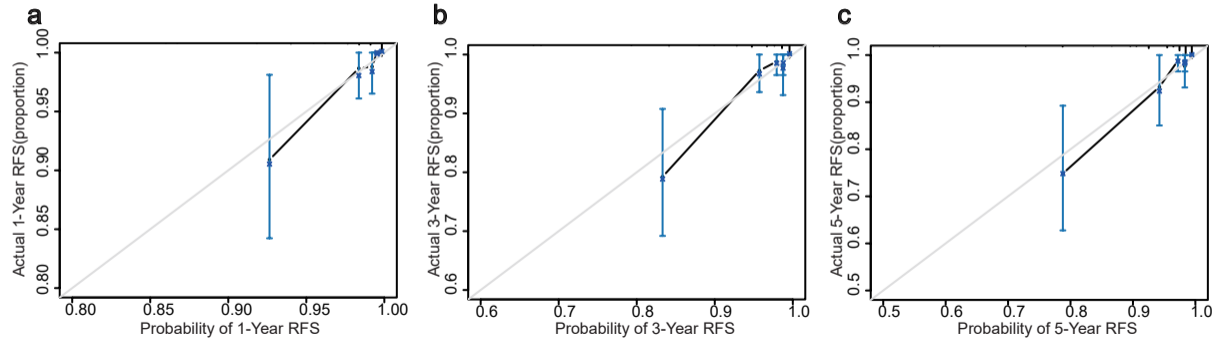

Supplement: Supplementary file 4 — Supplementary Figure S4. [file 41598_2023_47419_MOESM4_ESM.pdf]
