# Supplementary material for: Development and validation of a nomogram for predicting recurrence-free survival in endometrial cancer: a multicenter study
Source: Sci Rep. 2023 Nov 20;13:20270. doi: 10.1038/s41598-023-47419-8 (PMC10662280; doi:10.1038/s41598-023-47419-8)
Supplement: Supplementary file 6 — Supplementary Legends. [file 41598_2023_47419_MOESM6_ESM.docx]

**Fig. S1** The representative images of immunohistochemistry of P53 mutations. (a) The representative images of fully negative expression of P53 protein(40x); (b) The representative images of strongly positive expression of P53 protein(40x). P53 immunohistochemical antibody was purchased from ZSGB-BIO (Bei jing) with the item number of ZM-0408

**Fig. S2** The calibration curve of validation cohort from Tongji Hospital. (a) The calibration curve for the nomogram of predicting 1-year RFS in EC; (b) The calibration curve for the nomogram of predicting 3-year RFS in EC; (c) The calibration curve for the nomogram of predicting 5-year RFS in EC. EC: Endometrial cancer; RFS: Recurrence-free survival

**Fig. S3** The calibration curve of validation cohort from People’s Hospital of Peking University. (a) The calibration curve for the nomogram of predicting 1-year RFS in EC; (b) The calibration curve for the nomogram of predicting 3-year RFS in EC; (c) The calibration curve for the nomogram of predicting 5-year RFS in EC. EC: Endometrial cancer; RFS: Recurrence-free survival

**Fig. S4** The calibration curve of validation cohort from Qilu Hospital of Shandong University. (a) The calibration curve for the nomogram of predicting 1-year RFS in EC; (b) The calibration curve for the nomogram of predicting 3-year RFS in EC; (c) The calibration curve for the nomogram of predicting 5-year RFS in EC. EC: Endometrial cancer; RFS: Recurrence-free survival
